# Compensation Strategies for Gait Impairments in Parkinson's Disease: From Underlying Mechanisms to Daily Clinical Practice

**DOI:** 10.1002/mdc3.13616

**Published:** 2022-11-28

**Authors:** Anouk Tosserams, Bastiaan R. Bloem, Jorik Nonnekes

**Affiliations:** ^1^ Department of Rehabilitation, Center of Expertise for Parkinson & Movement Disorders, Donders Institute for Brain, Cognition and Behaviour Radboud University Medical Centre Nijmegen The Netherlands; ^2^ Department of Neurology, Center of Expertise for Parkinson & Movement Disorders, Donders Institute for Brain, Cognition and Behaviour Radboud University Medical Centre Nijmegen The Netherlands; ^3^ Department of Rehabilitation Sint Maartenskliniek Nijmegen The Netherlands

**Keywords:** Parkinson's disease, rehabilitation, compensation strategies, gait

## Introduction

Gait impairments are among the most disabling motor symptoms of Parkinson's disease (PD). These disturbances can be divided into two major groups, namely: continuously present gait disturbances; and episodically present gait disturbances. Continuous gait disturbances include a reduced step length and height, reduced gait speed, increased gait variability, stooped posture and asymmetrically reduced arm swing.^1^ Episodic gait disturbances include festination,^2^ and freezing of gait, the latter being operationally defined as a brief, episodic absence or marked reduction of forward progression of the feet despite the intention to walk.^3^ As the disease progresses, gait impairment generally becomes increasingly more severe, markedly affecting a person's mobility, independence and quality of life, and causing falls and related injuries.^4^, ^5^, ^6^

While dopaminergic therapy may have some beneficial effects on gait speed and step length, pharmacological treatment alone rarely suffices to adequately ameliorate gait quality—a problem that worsens further with disease progression, presumably because non‐dopaminergic lesions start to increasingly dominate the underlying pathophysiology.^7^, ^8^ Therefore, complementary non‐pharmacological interventions form an essential part of the management of gait impairment in PD. The contents of these non‐pharmacological interventions should be tailored to the individual patient, including the person's disease severity and ‐duration, and the specific type of gait disturbance(s).^9^, ^10^

In this review, we focus on one specific element within the broad spectrum of non‐pharmacological interventions for gait impairment in PD: the application of compensation strategies. The term compensation strategies refers to a wide variety of “detours” that are typically spontaneously invented by persons with PD to improve their gait. Examples of such strategies include: walking while rhythmically counting, while bouncing a ball, or while mimicking the gait pattern of another person.^11^ Such strategies were initially reported mainly in the form of anecdotal case reports,^12^, ^13^, ^14^, ^15^ but the body of literature on compensation strategies for gait impairment in PD has grown substantially in recent years, including more robust, systematic investigations into their efficacy and potential underlying mechanisms.

Here, we provide a comprehensive overview of the current knowledge on compensation strategies for gait impairment in PD. We cover reports on the objectively measured (lab‐based) efficacy, as well as the subjective (patient‐rated) efficacy of compensation strategies in improving gait in PD, and discuss the suspected mechanisms underlying the various forms of compensation. Moreover, we will provide concrete recommendations for clinical practice based on the presented evidence, and share suggestions for future investigations on this fascinating topic.

### Classification of Compensation Strategies

In this review we will refer to seven distinct categories of compensation strategies. This categorization is based on the first‐ever large‐scale review of the available compensation strategies for gait impairment in PD, which was published in 2019.^11^ Over a period of 4 years, the authors of this review collected several hundred videos of patients who used self‐invented tricks and aids to improve their mobility in daily life. Analysis and subsequent classification of this footage yielded 59 unique compensation strategies, that all appeared to fit into one of several basic compensatory concepts. The authors proposed a classification of these strategies, based on their suspected underlying working mechanisms. The seven categories entailed: (1) external cueing; (2) internal cueing; (3) motor imagery and action observation; (4) altering the mental state; (5) changing the balance requirements; (6) adopting a new walking pattern; and (7) alternatives to walking. Table 1 presents an overview of this classification of compensation strategies for gait impairments in PD, including practical examples of strategies, and the prevailing hypotheses regarding their principal underlying mechanisms.

**TABLE 1:** Classification of compensation strategies for gait impairments in PD.^11^

Compensation strategy	*Principal mechanism*	*Phenomenology*
External cueing	Introduction of goal‐directed behavior by introducing a movement reference or target	Walking to the rhythm of music; Stepping over lines on the floor; Bouncing a ball
Internal cueing	Assist in achieving focused attention towards specific components of gait, to shift from automatic to goal‐directed motor control	Mental singing or counting; Focusing on a specific component of the gait cycle (e.g. making a heel strike)
Changing the balance requirements	Facilitate the ability to make lateral weight shifts, thereby easing the swing phase of the unloaded leg, particularly in gait initiation or turning.	Using walking aids; Making a volitional weight shift before gait initiation; Making wider turns.
Altering the mental state	Enhance general alertness and arousal. This may help shift from automatic to goal‐directed motor control	Reducing anxiety (e.g. mindfulness); Increasing motivation (e.g. encouraging oneself); Kinesia paradoxa
Motor imagery and action observation	Activate the mirror neuron system which may facilitate cortically generated movement.	Observing or visualizing and mimicking the gait pattern of another person.
Adopting a new walking pattern	Use alternate motor programs that may be less overlearned and less dependent on the automatic mode of motor control.	Skipping; Walking backwards or sideways; Running; Making skating movements.
Alternatives to walking	Walking difficulty may be a task‐specific problem	Riding a bicycle; Skateboarding; Riding a scooter; Roller skating

*Note*: Adapted from reference.^11^

## Evidence of the Efficacy of Compensation Strategies

Over the last decades, several large trials have investigated the efficacy of compensation strategies for gait impairments in PD, with a clear emphasis on external cueing strategies (i.e. visual, auditory or tactile cues). The remaining categories of strategies remain to be explored further, because they are still relatively unknown (e.g. motor imagery), or because they are rather difficult to control in a lab‐based setting (e.g. altering the mental state). Furthermore, especially in earlier studies on the efficacy of cueing strategies, the focus was primarily on alleviating freezing of gait (the main example of an episodic gait disturbance). However, compensation strategies are equally useful in ameliorating continuous gait disturbances (e.g. correcting the reduction in step length, or reducing gait variability).^16^ A complete overview of the available evidence on the efficacy of all available compensation strategies is beyond the scope of this review. Instead, we will focus on a selection of trials that have markedly influenced the field.

A systematic review and meta‐analysis of 50 studies (*n* = 1892 subjects) on the efficacy of (external) auditory cueing concluded that auditory cueing improves gait quality in persons with PD.^17^ This analysis revealed that 88% of the included studies reported beneficial effects of auditory cueing on spatiotemporal gait parameters, including stride length, and gait speed. Another meta‐analysis of 28 studies (*n* = 718 subjects) on the efficacy of (external) auditory versus visual cues also demonstrated that both modalities increased stride length, and improved gait speed in persons with PD.^18^ External cues can also improve gait initiation in persons with PD, through enhanced anticipatory postural adjustment.^19^, ^20^ Notably, the efficacy of cueing strategies has predominantly been investigated in single‐session experiments in lab‐based settings. However, in the RESCUE trial,^21^ a single‐blind, randomized clinical trial including 153 persons with PD, participants received a 3‐week home‐based cueing program using a multimodal (external) cueing device. The RESCUE trial demonstrated modest, but significant improvements in gait with cues, including increased gait speed and step length, as well as a reduction in freezing of gait severity among freezers. Cueing generally showed an immediate correction of gait, with limited carry‐over or training effects to uncued gait performance post‐intervention.^21^, ^22^ This underlines the notion that compensation strategies should likely be applied on‐demand throughout the day, and that intermittent training alone is not sufficient.

Limited studies have been performed to investigate the efficacy of the remaining categories of compensation strategies for gait impairment in PD. Several studies have established the efficacy of internal cueing, for example in the form of mental singing while walking, to reduce gait variability in PD.^23^, ^24^, ^25^ Voluntary lateral weight shifting (part of the category ‘changing the balance requirements’) was demonstrated to support a more effective gait initiation,^26^ as well as more effective turning in persons with PD who manifested freezing of gait.^27^ For action observation, a randomized clinical trial revealed a significant reduction in bradykinesia during a finger tapping task,^28^ but the effects on gait in PD should be explored further. Motor imagery was previously found to be a successful strategy in normalizing stride length in persons with PD.^29^ Exaggerating the arm swing during gait (part of the category “adopting a new walking pattern”) has been demonstrated to improve gait initiation,^30^ as well as increase gait speed and step length.^31^ Finally, the efficacy of “altering the mental state” strategies is—thus far—predominantly based on anecdotal reports of kinesia paradoxa: “The sudden, transient ability of a person with PD to perform a task they were previously unable to perform“.^32^ A classic example includes the account of the 2009 L'Aquila earthquake, during which severely affected persons with PD were able to independently escape from the collapsing buildings, helping relatives on their way out.^33^ It remains to be established how persons with PD could make optimal use of this phenomenon in non‐threatening, daily‐life situations.

## Perceptions of Compensation Strategies

Besides clinical trials aiming to quantify the objective effects of compensation strategies on gait quality in persons with PD, recent studies have also focused on the subjective perceptions of these strategies among both persons with PD and their healthcare providers. We will next discuss these studies in further detail.

### Persons with Parkinson's Disease

In 2021, a large survey study on the perceptions and use of compensation strategies among 3243 persons with PD and gait impairments within the Michael J. Fox Foundation Fox Insight (USA) and ParkinsonNEXT (NL) cohorts was published.^16^ Each of the seven categories of compensation strategies was explained, and participants were asked if they were aware of these strategies, if they had ever used such strategies, and if so, what the efficacy of these strategies was in a variety of contexts (e.g. gait initiation, turning, stopping, crossing a doorway, walking outdoors).

The study revealed that the patients’ overall knowledge of the broad spectrum of available strategies is rather limited. While the self‐reported severity of gait impairment among participants was relatively high (i.e. 35% claimed that this affected their ability to perform usual daily activities, and 52% had fallen at least once in the past year), one in five patients had never heard of any form of compensation strategies before. Only 4% of participants were aware of all seven categories of compensation strategies. In line with a related awareness study among PD healthcare professionals,^34^ the best‐known strategies among persons with PD were external and internal cueing, which were known to just under half of the participants. The least known category was action observation and motor imagery.

Approximately 65% of respondents used one or more compensation strategies in daily life at the time of the survey. Compensation strategies were most often used when walking outdoors, or in time–pressure situations, and were least often applied when attempting to stop walking or cross a doorway. Changing the balance requirements (e.g. making a volitional weight shift to initiate gait) was the most widely used category, followed by internal cueing. Notably, while external cueing was the best‐known category among persons with PD, it was applied least often in daily life by persons with PD. External cueing may be less accessible or feasible than other types of strategies, as it typically requires adaptations to the environment (e.g. 2‐ or 3D patterns on the floor), or specific devices (e.g. laser shoes, a metronome). External cues may also be less preferred because of their visibility to bystanders, thereby causing stigmatization or feelings of embarrassment.^35^, ^36^

Overall, the patient‐reported efficacy of all categories of compensation strategies was high. Changing the balance requirements was most often reported to have a beneficial effect on gait (76% of patients that had ever tried it found it had a positive effect), whereas external cueing showed the relatively lowest success rate (62%).

### Healthcare Professionals

A survey study on the perceptions of compensation strategies for gait impairment in PD was conducted among 320 PD healthcare professionals in the Netherlands.^34^ PD healthcare professionals (i.e. physical therapists, occupational therapists, specialized PD nurses, general nurses, and movement disorders specialists) who saw at least one person with PD per month in their clinical practice received a summary of each of the seven categories of compensation strategies, illustrated by practical examples. They were then queried about their previous awareness of these strategies, and whether they had ever applied them in their clinical practice when working with persons with PD and gait impairments.

Only 35% of the professionals were aware of the existence of all seven categories of compensation strategies, and 23% applied strategies from all categories in practice. Of all available strategies, external and internal cueing paradigms were most often applied (by 94%, and 93% of respondents, respectively). Action observation and motor imagery was the least known category among professionals, and was applied in clinical practice by less than half of the respondents. Importantly, most professionals indicated that a lack of specific knowledge and skills concerning certain categories of compensation strategies was the main reason why they did not include strategies from all seven categories in their daily practice. Considering the study design—which included a high risk of selection bias—and the fact that it was conducted in the Netherlands, which has organized PD care in a high‐standard national network of specifically trained (allied) healthcare professionals (ParkinsonNet^37^), the actual knowledge of compensation strategies for gait impairment in PD among healthcare professionals across the globe may even be considerably more limited.

## Mechanisms Underlying Compensation Strategies

The efficacy of compensation strategies for gait impairments in PD has now been established, but their exact underlying mechanisms remain relatively unclear. The study of the mechanisms underlying compensation strategies is a rapidly emerging field. Dynamic neuroimaging techniques such as electroencephalography (EEG), and functional near‐infrared spectroscopy (fNIRS) now allow us to study the cortical mechanisms of compensation strategies during actual gait, rather than imagined gait (to avoid movement artifacts) while the participant is lying in a scanner. Improving our understanding of the key mechanisms underlying compensation at the neurological systems level will eventually facilitate the development of innovative rehabilitation interventions to improve gait in persons with PD. Here, we will discuss the prevailing hypotheses in the field regarding the primary working mechanisms of compensation strategies for gait impairment in PD, as well as some very recent, preliminary insights from studies using dynamic neuroimaging techniques.

### Automated Vs. Goal‐Directed Behavior

The pathophysiology underlying gait impairments in PD is complex and presumably involves dysfunction of multiple cortical and subcortical components within the locomotor network. Gait partly depends on a basic “locomotor network”, involving spinal central pattern generators, brainstem mesencephalic and cerebellar locomotor regions, and corticostriatal input projecting to the primary motor cortex.^38^ In addition, distributed cortical areas, particularly the frontoparietal and supplementary motor areas, are involved in adjustment and adaptation of gait. During walking in an automated manner, persons with PD have difficulties recruiting cortical motor areas.^39^ Indeed, persons with PD generally experience more difficulties when walking in an automated manner (i.e. without consciously paying attention to it), compared to when producing goal‐directed behavior (often facilitated by the presence of a clear external stimulus or “cue”).^40^ These differences between automatic and goal‐directed behavior in PD are likely related to a greater loss of dopaminergic innervation in the posterior putamen, which has been associated with the control of automatic (habitual) behavior, in contrast to the relatively preserved rostromedial striatum, which is primarily involved in goal‐directed behavior.^41^, ^42^ Consequently, persons with PD may increasingly have to rely on making a compensatory shift from the automated to the goal‐directed mode of action control to maintain functional mobility. The application of compensation strategies is believed to facilitate this shift from automated to goal‐directed gait control.^11^

### Use of Alternative Pathways to Control Gait

Recently, the cortical correlates of external auditory cueing, internal cueing and action observation were investigated in 18 persons with PD that had previously shown a beneficial response to these three strategies in a controlled lab‐setting.^43^ High‐density EEG was recorded both during stance and gait on a treadmill under four conditions: (1) without strategies; (2) with external cueing (listening to a metronome); (3) with internal cueing (silent rhythmic counting); and (4) with action observation (observing another person walking). The application of compensation strategies resulted in changed cortical activity compared to baseline gait, which could not be solely attributed to stimulus‐related sensory processing (e.g. auditory processing of the metronome sound, or visual processing during action observation). Relative to baseline gait, the use of all three compensation strategies induced increased cortical activation of the sensorimotor areas. Furthermore, cortical activation patterns differed depending on the type of compensation strategy that was applied, suggesting that each of the strategies engages a distinct cortical network to achieve enhanced central motor activation in persons with PD, and that there are multiple “routes” to control gait.^43^ These findings support the prevailing hypothesis that central motor activation could be achieved through cues by making use of alternative pathways to control gait.^10^, ^11^, ^44^

Interestingly, in contrast to prior beliefs, recent observations suggest that frontal attentional and executive brain regions do not seem to play a major role in the mechanisms underlying external cueing. An ambulatory EEG study in 20 healthy controls and 43 persons with PD found that gait with external visual cues did not significantly influence frontal brain activity.^45^ Similarly, an fNIRS pilot study in 25 persons with PD showed that the use of external somatosensory cues did not increase activation of the prefrontal cortex compared to uncued gait.^46^ Rather than eliciting increased frontal activation, external cues primarily elicit parieto‐occipital activation, and seem to introduce goal‐directed motor control by providing a movement reference or target.^11^, ^43^ Frontal regions do, however, seem to be relevant in internal cueing, as demonstrated by increased frontal activation during the application of rhythmic mental counting during gait (which could not be attributed to frontal activation elicited by the execution of a rhythmic mental counting task alone).^43^ Indeed, internal cues have been hypothesized to assist in filtering and prioritizing tasks, and achieving focused attention on specific elements of gait.^11^

## Evaluation of Compensation Strategies in Clinical Practice

The fact that different types of compensation strategies seem to rely on distinct cortical networks could potentially explain the inter‐individual variation in the efficacy of compensation strategies.^43^ While a specific strategy may work well for one person, it could have no effect, or may even aggravate gait impairments in another person, depending on the specific extent of the neuropathological changes in the brain for each affected individual.^16^ A one‐size‐fits‐all approach to the use of compensation strategies for PD gait rehabilitation therefore does not apply. Thus far, it has not been possible to predict which strategies would suit an individual patient best based on specific patient characteristics (e.g. age, sex, disease duration, cognitive status, or presence of freezing of gait).^16^ However, in a recent prospective study on compensation strategies in a cohort of 101 persons with PD and gait impairment, certain patient characteristics were associated with larger improvements in gait variability using compensation strategies (*submitted work*). Participants without freezing of gait, with lower MDS‐UPDRS scores and greater balance capacity, showed the largest improvements, implying that a certain level of functional reserve seems necessary to optimally benefit from the use of compensation strategies for gait impairment in PD.

Besides inter‐individual differences in the efficacy of specific strategies, the efficacy of a strategy typically also depends on the context or situation in which it is applied. For example, 73% of persons with PD perceived the use of internal cues to be helpful during gait initiation, but only 47% found it helpful when attempting to stop walking.^16^ These findings further emphasize the need for an individually tailored, personalized approach to the use of compensation strategies. In Table 2 we provide four practical tips for evaluating compensation strategies in clinical practice, based on our own clinical experience.

**TABLE 2:** Four practical tips for evaluating compensation strategies in clinical practice

Determine the primary element(s) of gait you wish to improve
Consider your patients personal preferences, and make creative use of their hobbies or skills
Evaluate the strategy in the context in which it will eventually be used in daily life
Get inspired by patient videos of available strategies on www.walkingwithparkinson.com

## Future Directions

Compensation strategies are increasingly recognized as an essential element of gait rehabilitation in PD, generating novel systematic scientific endeavors to investigate their practical applications in daily clinical practice. Besides external cueing paradigms, other forms of compensation strategies also deserve further attention, and future clinical trials should be aimed at mapping the inter‐individual differences between responders and non‐responders to certain categories of strategies, aiming to eventually work towards a more personalized, tailored approach to gait rehabilitation. We will address two recommendations for further inquiries regarding: (1) the implementation of such a personalized approach to gait rehabilitation in primary care practices; and (2) a plan of action to further explore the exact underlying mechanisms of these strategies at brain network level.

### Towards Accessible, Personalized Gait Rehabilitation in Parkinson's Disease

In this review, we established that persons with PD generally require multiple strategies that are specifically tailored to their unique needs and circumstances in order to perform their daily activities.^16^ Ideally, persons with PD should have access to personalized gait rehabilitation close to home, in local primary‐care physiotherapy practices, rather than in university hospitals spread across the country. To facilitate the personal search for suitable strategies, it is crucial that healthcare professionals have adequate knowledge about the wide variety of strategies. Yet, we also noted that the knowledge regarding compensation strategies for gait impairment in PD is suboptimal among physiotherapists.^34^ Therapists indicate that they lack a comprehensive oversight, that they do not use a systematic approach, and that they would ideally like to be able to consult an expert when needed. To overcome these barriers, healthcare innovations bridging specialized PD care with primary care practices should be explored. For example, by empowering primary care physiotherapists in using a systematic, personalized approach to the evaluation of compensation strategies, with the ability to digitally consult remote experts for on‐demand support. Such a remote support strategy would be particularly attractive in loosely populated areas of the world where access to specialized care is more difficult.

### Towards a Better Understanding of the Underlying Mechanisms of Compensation Strategies

As we discussed earlier, the underlying mechanisms of compensation strategies have been explored using dynamic neuroimaging techniques including EEG and fNIRS during actual gait in PD.^43^, ^45^, ^46^ This way, we now have a general understanding of the cortical correlates of these gait strategies. However, in order to obtain a more complete picture, future studies should aim to combine the advantages of different methodologies. For example, by using a multi‐modal approach, combining EEG (dynamic, high temporal resolution), functional MRI (high spatial resolution), diffusion tensor imaging (to map connectivity), concurrently delivered transcranial magnetic stimulation and EEG (to measure cortical excitability), and neuromodulation using transcranial alternating current stimulation (to assess neural plasticity). Furthermore, contrasting the brain responses of responders (persons who benefit from a certain strategy) to non‐responders (persons who do not) may also provide valuable insights into the underlying mechanisms of gait compensation strategies in PD.

## Conclusion

We hope that this overview may serve as a preface for a new journey of discovery regarding the underutilized potential of compensation strategies to improve gait in persons with PD. Raising awareness about the full spectrum of available strategies among people with PD and healthcare professionals, and gaining more insight into the determinants of inter‐individual differences in response to these strategies, as well as their exact underlying mechanisms, will ultimately pave the way towards a more personalized approach to PD gait rehabilitation.

## Author Roles

(1) Research project: A. Conception. (2) Manuscript Preparation: A. Writing of the first draft, B. Review and Critique.

AT: 1A, 2A.

BB: 1A, 2B.

JN: 1A, 2B.

### Disclosures

**Ethical Compliance Statement:** We confirm that we have read the journal's position on issues involved in ethical publication and affirm that this work is consistent with those guidelines. The authors confirm that approval of an institutional review board and patient consent were not required for this work.

**Funding Sources and Conflicts of Interest:** Jorik Nonnekes was supported by a ZonMW Veni grant (16.196.022). The Center of Expertise for Parkinson & Movement Disorders was supported by a center of excellence grant awarded by the Parkinson Foundation. The authors declare that there are no conflicts of interest relevant to this work.

**Financial Disclosures for the Previous 12 Months: AT reports no disclosures.** JN was supported by a ZonMW Veni (grant 16.196.022). He reports a grant from ZonMW (OffRoad grant), Michael J. Fox Foundation, Ipsen Pharmaceuticals and Gossweiler Foundation unrelated to the present work. He serves at the scientific advisory board of Cue2Walk and Ceriter. BB currently serves as co‐Editor‐in‐Chief for the Journal of Parkinson's disease, serves on the editorial board of Practical Neurology and Digital Biomarkers, has received honoraria from serving on the scientific advisory board for the Critical Path Institute, Kyowa Kirin, UCB and Zambon, has received fees for speaking at conferences from AbbVie, Bial, GE Healthcare, Oruen, Roche and Zambon, and has received research support from the Netherlands Organization for Scientific Research, the Michael J. Fox Foundation, Nothing Impossible, Parkinson Vereniging, the Parkinson's Foundation, Hersenstichting Nederland, Verily Life Sciences, Horizon 2020, the Topsector Life Sciences and Health, Abbvie, UCB, Yesse Technologies and Zambon, all unrelated to the present work.
